# Electro-Biocatalytic Reactivity of Catecholamine at a Lignin Nanoparticle–Tyrosinase Interface

**DOI:** 10.3390/molecules31162839

**Published:** 2026-08-14

**Authors:** Valeria Gigli, Elisabetta Tomaino, Davide Piccinino, Lorenzo Botta, Eliana Capecchi, Raffaele Saladino

**Affiliations:** 1 Research Centre for Innovation in Circular Economy and Sustainability (CRIECS), University of Tuscia, 02100 Rieti, Italy; valeria.gigli@unitus.it; 2 Department of Biological and Ecological Sciences, University of Tuscia, 01100 Viterbo, Italy; e.tomaino@unitus.it (E.T.); lorenzo.botta@unitus.it (L.B.); 3 Centro Integrato di Ateneo, Sezione Grandi Attrezzature, University of Tuscia, 01100 Viterbo, Italy; d.piccinino@unitus.it; 4 Department of Chemistry and Drug Technologies, Sapienza University of Rome, 00185 Rome, Italy; raffaele.saladino@uniroma1.it

**Keywords:** biocatalytic control, sustainable electrocatalysis, catecholamines detection, bioconjugates, electroactive lignin nanoparticles

## Abstract

The electrochemical sensing of similar catecholamines remains challenging due to their overlapping redox behavior and similar reactivity, which often results in poorly selective reaction pathways. Herein, we report a bioinspired tyrosinase electro-catalytic system that enables the modulation of the catecholamine reactivity through the integration of enzymatic oxidation with electrochemical transformation. The biocatalytic platform consisted of electroactive lignin nanoparticles (LNPs) supporting tyrosinase drop cast on the graphene-based screen-printed electrode. The overall reaction included the oxidation of catecholamines to ortho-quinones, followed by nucleophile addition of cysteine under control of the redox environment. Overall, coupling enzymatic catalysis with electrochemical regulation enabled selectivity in the oxidative functionalization of catecholamines, providing a sustainable strategy for tuning reactivity in advanced bioinspired catalytic systems.

## 1. Introduction

The development of rapid and selective methods for the early diagnosis of disorders associated with neurotransmitter imbalance is of growing importance [1]. However, the selective recognition of structurally related catecholamines, such as dopamine, epinephrine and norepinephrine, remains challenging due to their structural similarity and closely overlapping redox behavior, which often leads to poorly resolved electrochemical responses [2]. An alternative strategy relies on the transformation of similar substrates into distinct chemical entities. In this context, approaches based on the in situ formation of bioconjugates offer a powerful tool for in situ modulation of molecular structure [3,4]. Catecholamines are known to undergo oxidation to corresponding ortho-quinones, that are reactive intermediates in biological systems [5]. Quinones readily react with nucleophilic species such as cysteine and other thiols, leading to a wide variety of conjugates [6]. In particular, the oxidation of dopamine to quinones enables protein binding and polymerization pathways in the formation of neuromelanin [6]. Notably, the addition of thiols to ortho-quinones displays unusual and still debated regio-chemistry, often deviating from that expected for classical Michael-type processes [7]. These reactions may proceed through radical mechanisms involving semiquinone and thiyl radical intermediates, rather than purely ionic pathways [7,8]. The coexistence of multiple catecholamine-derived metabolites within neuromelanin highlights the intrinsic tendency of these systems to generate complex and heterogeneous networks [8]. Such transformations can therefore generate chemically distinct species that contribute to substrate-dependent redox-active ensembles. The use of thiol-mediated reaction networks to enable electrochemical differentiation of catecholamines is not explored, particularly in the case of hybrid bio-based interfaces. Recently, electrochemical methods emerged as powerful tools for monitoring the release of catecholamine at high temporal and spatial resolution, as well as for organic electrosynthesis applications [9]. Herein, we report a novel cysteine-driven reaction occurring at the lignin nanoparticle–tyrosinase interface, in which enzymatic oxidation and nucleophilic conjugation of catecholamines with cysteine act in a concerted way to generate substrate-dependent redox ensembles. In these transformations, tyrosinase acts as a diphenolase, enabling the two-electron oxidation of catecholamines to reactive ortho-quinones [10]. Previous examples of the use of immobilized tyrosinase on lignin nanoparticles have been reported in the coupling of phenols and in the nucleophile-triggered cascade of bicyclic and tricyclic benzoxazines [11,12]. The present electro-biocatalytic system is proof-of-concept about the possibility to use differential pulse voltammetry and enzymatic transformation for the control of the reaction of dopamine, epinephrine, and norepinephrine with L-cysteine. HPLC–MS and HRMS analyses confirmed the formation of mono-cysteinyl adducts under the reported experimental conditions.

## 2. Results

### 2.1. Preparation and Characterization of Lignin Nanoparticles from Enzymatic Hydrolytic Lignin

**Figure 1:**
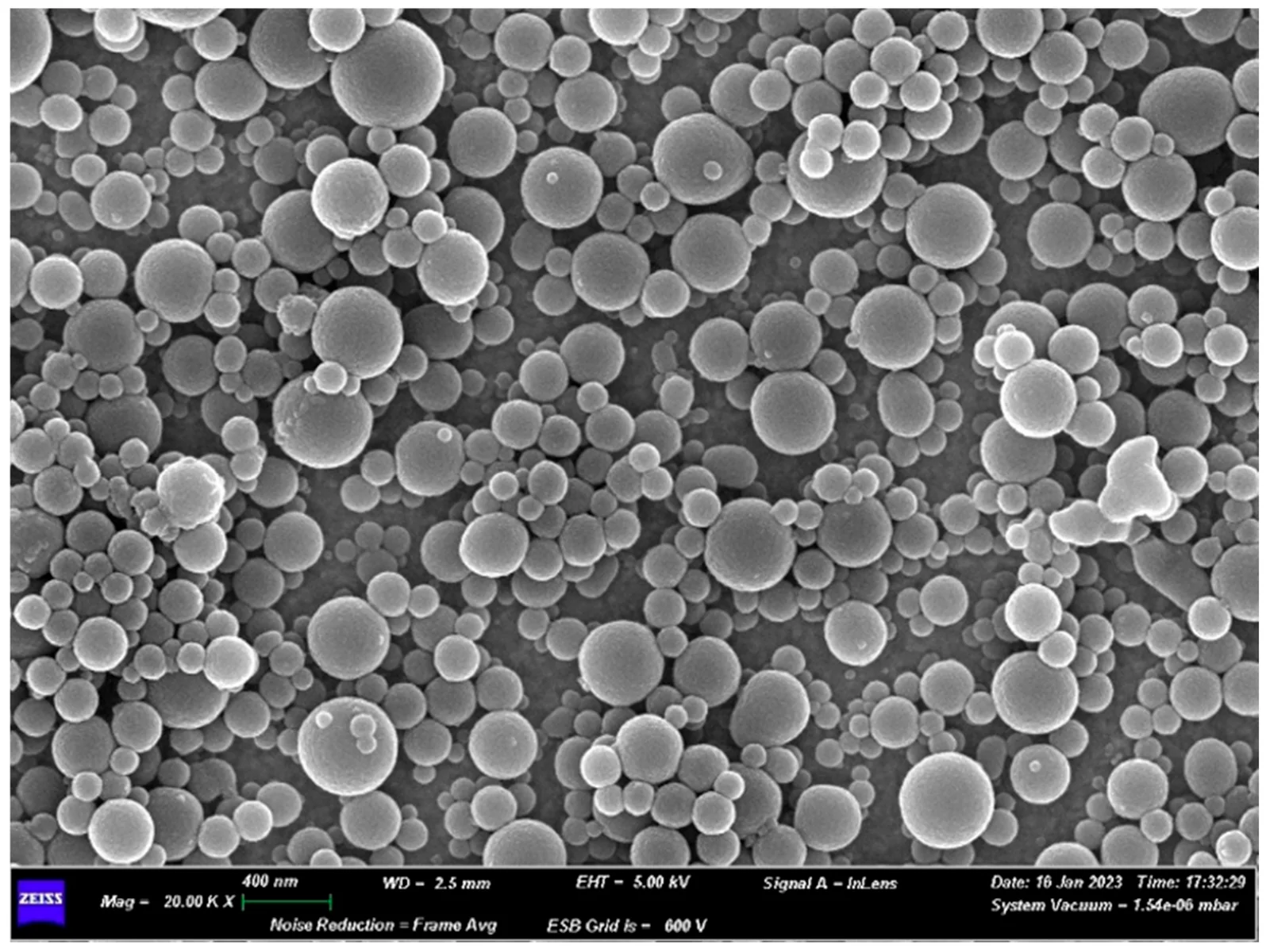
FE-SEM image of EHLNPs.

Lignin nanoparticles (LNPs) were prepared from commercially available enzymatic hydrolytic lignin (EHL) [13]. The distribution and variety of hydroxyl group in EHL was preliminarily determined by quantitative ^31^P NMR analysis after phosphitylation reaction with 2-chloro-4,4,5,5-tetramethyl-1,3,2-dioxaphospholane (TMDP) and it is reported in Table S1 [14]. EHL was characterized by a high content of para-hydroxyphenyl OH groups which are expected to modulate electron transfer properties [15]. Lignin nanoparticles from EHL (EHLNPs) were obtained by application of sustainable nanoprecipitation technology [15], encompassing dissolution of lignin in a ternary mixture composed by water, ethanol, and THF and successive addition of deionized water as antisolvent. EHLNPs were characterized by dynamic light scattering (DLS), showing an average hydrodynamic diameter of 390 nm and low polydispersity index (PDI = 0.1), with a narrow size distribution and ζ-potential of −56.95 mV, consistent with stable colloidal dispersion (original DLS analysis is in Figure S1). Morphological characterization by field-emission scanning electron microscopy (FE-SEM) confirmed the formation of spherical nanoparticles (Figure 1).

### 2.2. Electrochemical Performance of Screen-Printed Electrode Platforms

**Figure 2:**
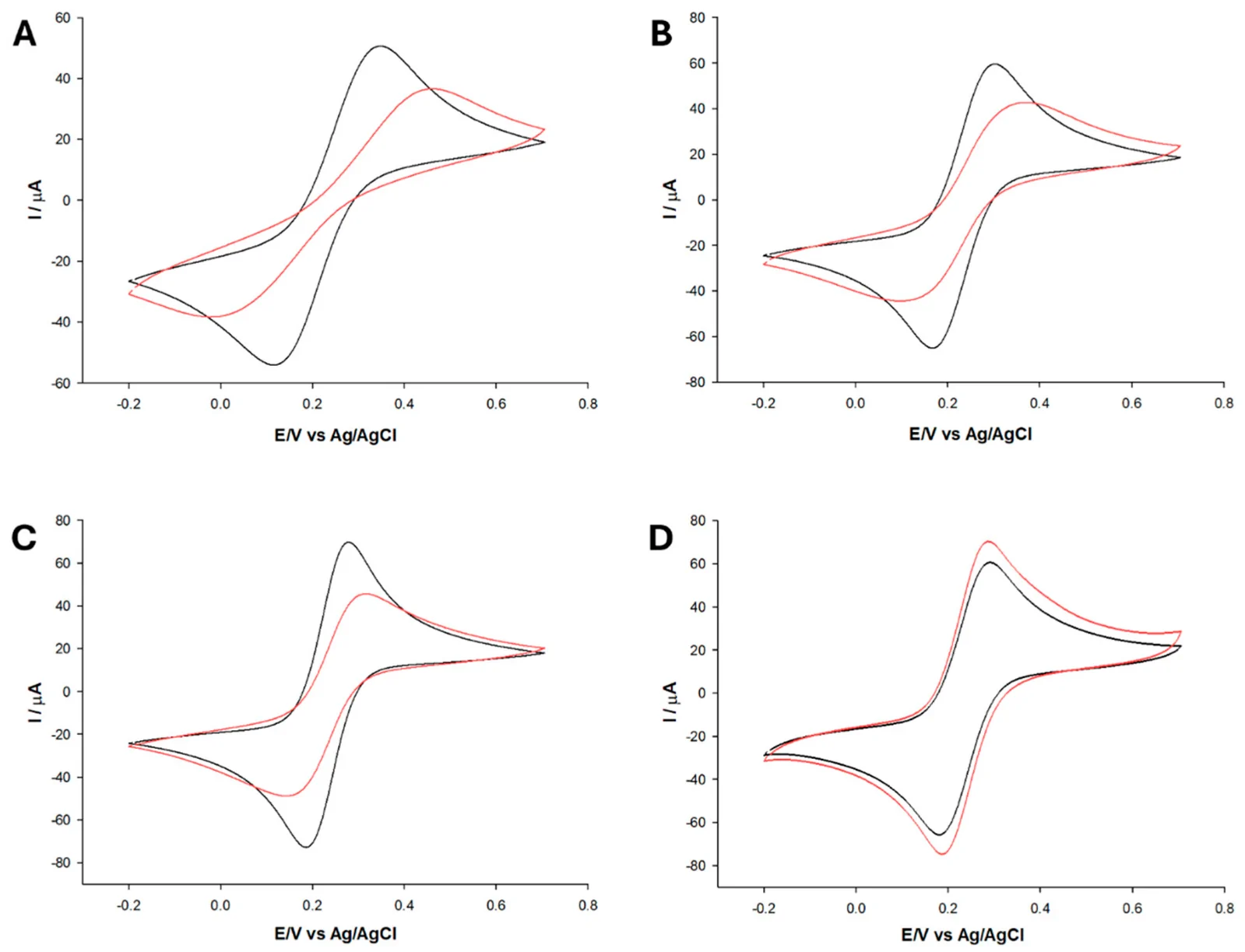
CV of different screen-printed electrode (SPE) platforms recorded in a 2.5 mM [Fe(CN)_6_]^3−^/^4−^ solution containing 0.1 M KCl, at a scan rate of 25 mV s^−1^: (**A**) SPCE, (**B**) MWCNTs/SPE, (**C**) Au-SPE, and (**D**) GPH/SPE. The black line represents the bare electrode and the red line the EHLNPs modified electrode.

The electrochemical performance of different screen-printed electrode (SPE) platforms, both bare and modified with EHLNPs, was investigated by cyclic voltammetry (CV) using the [Fe(CN)_6_]^3−^/^4−^ redox probe (Figure 2). The comparison among screen-printed carbon electrode (SPCE) (Figure 2A), multi-walled carbon nanotubes screen-printed electrode (MWCNTs/SPE) (Figure 2B), gold screen-printed electrode (Au-SPE) (Figure 2C), and graphene-based screen-printed electrode (GPH/SPE) (Figure 2D) showed significant differences in electron transfer kinetics and surface properties, as reflected by the peak-to-peak separation (ΔE) and peak currents (Table 1, entries 1–8). The ΔE value of 59 mV at 25 °C is expected for the diffusion-controlled reversible one-electron redox couple with [Fe(CN)_6_]^3−^/^4−^, whereas larger ΔE values indicate increasing deviations toward quasi-reversible behavior or kinetically hindered electron transfer processes [16].

Among the bare electrodes, Au-SPE and GPH/SPE showed the best performance (Table 1, entries 3 and 4, respectively) and highest currents. In contrast, SPCE was characterized by the poorest response (Table 1, entry 1), probably because of the occurrence of slow and less reversible electron transfer processes. MWCNTs/SPE displayed intermediate behavior (Table 1, entry 2).

The bare SPE was subsequently modified by drop-casting with freshly prepared EHLNPs, yielding a nanostructured lignin interfacial layer. Following modification, most platforms showed a general increase in ΔE (Figure 2A–D), suggesting the partial hindrance of electron transfer and shift toward irreversible behavior. This effect was particularly evident in the case of EHLNPs/SPCE (Figure 2A), where ΔE increased to 392 mV (Table 1, entry 5) [15]. A similar, though less pronounced, trend was observed for EHLNPs/MWCNTs/SPE (Figure 2B; Table 1, entry 6) and EHLNPs/Au-SPE (Figure 2C, Table 1, entry 7). The modification of GPH/SPE with EHLNPs (Figure 2D) did not affect the electrochemical response, maintaining a low value of ΔE (91 mV; Table 1, entry 8) and increasing peak currents compared to bare electrode (Table 1, entry 4).

Overall, EHLNPs/GPH/SPE emerged as the most suitable electrochemical nanoplatform, combining high conductivity and low ΔE, in agreement with the previous literature reports [15,17,18]. EHLNPs/GPH/SPE was selected for the next steps.

### 2.3. Electrochemical Behavior of EHLNPs/GPH/SPE

**Figure 3:**
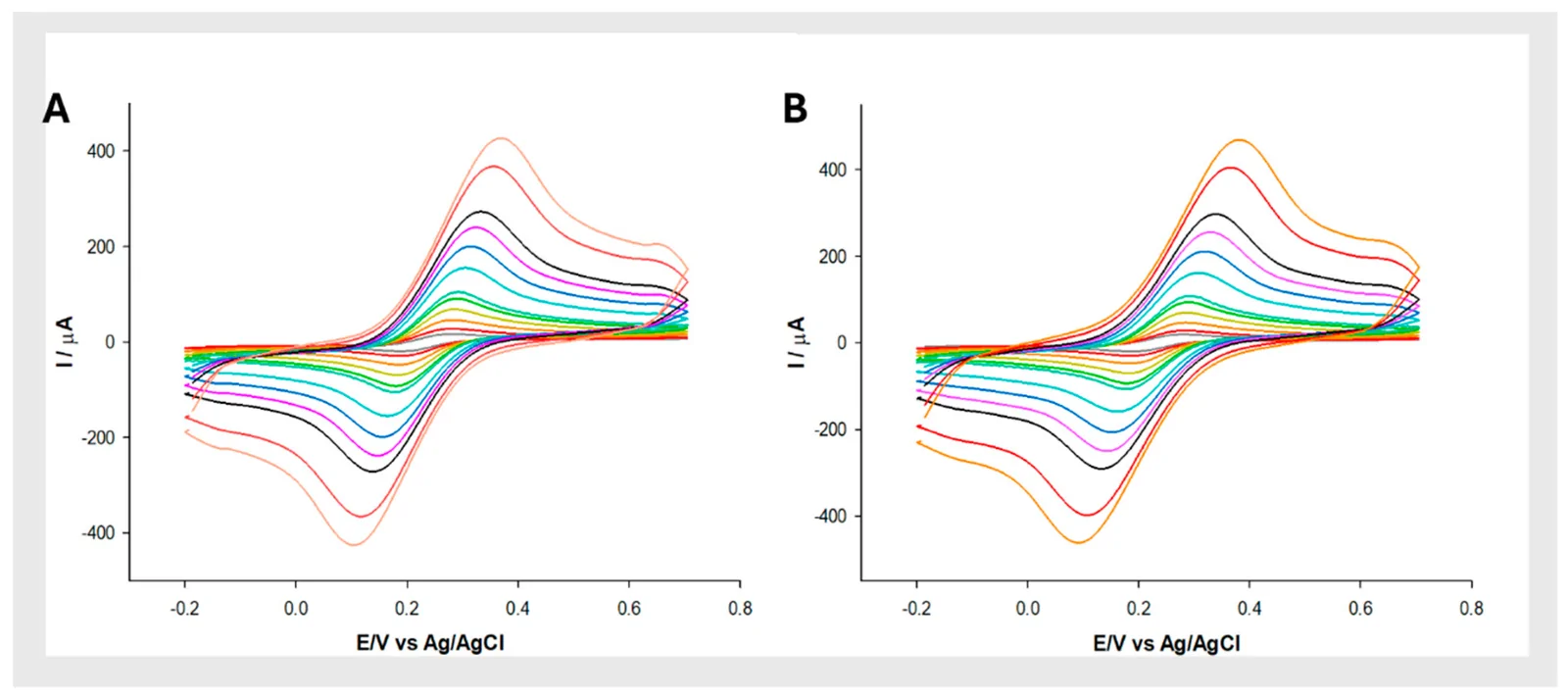
Effect of scan rate on the CV response of (**A**) GPH/SPE and (**B**) EHLNPs/GPH/SPE in a 2.5 mM [Fe(CN)_6_]^3−^/^4−^ solution containing 0.1 M KCl. Scan rates: 5–1000 mV s^−1^. The different color represent the CVs at different scan rate.

The electrochemical behavior of GPH/SPE before and after modification with EHLNPs was further investigated by CV at different scan rates (from 5 mV s^−1^ to 1000 mV s^−1^) using 2.5 mM [Fe(CN)_6_]^3−^/^4−^ redox probe solution (Figure 3A,B). The electroactive area (A_E_), the roughness factor (ρ), and the heterogeneous electron transfer rate constants (k_0_), is summarized in Table 2. The A_E_ was calculated from the slope of the Ip vs. v^1/2^ plots according to the Randles–Sevcik equation [19]:

(1) $$I_{p}=2.686\times 10^{5\;}n^{3/2}A_{E}D_{0}^{1/2}C_{0}v^{1/2}$$

where I_p_ is the peak current (A), *n* is the number of electrons involved in the redox process, A_E_ is the electroactive area (cm^2^), D_0_ is the diffusion coefficient (7.6 × 10^−6^ cm^2^ s^−1^ for ferricyanide), C_0_ is the bulk concentration (mol cm^−3^), and *v* is the scan rate (V s^−1^).

The A_E_ increased from 0.19 cm^2^ for GPH/SPE to 0.22 cm^2^ for EHLNPs/GPH/SPE, indicating a slight improvement of the surface available for electron transfer upon modification with lignin nanoparticles. The roughness factor (ρ), defined as the ratio between the electroactive and geometric areas, increased from 1.74 to 2.04 because of improved surface heterogeneity of the electrode interface. The value of k_0_ was determined using the approach proposed by Lavagnini et al., which combines the models developed for irreversible systems (Klingler–Kochi) with those for reversible ones (Nicholson and Shain) [20]. A slightly higher k_0_ value was obtained for the EHLNPs/GPH/SPE compared to the bare.

Overall, the observed increase in A_E_, ρ, and k_0_ suggests that the EHLNPs layer does not act as an insulating barrier but rather slightly enhances the electroactive surface and the charge transfer properties of the electrode.

### 2.4. Electrochemical Behavior of Catecholamines at the EHLNPs/GPH/SPE Interface

**Figure 4:**
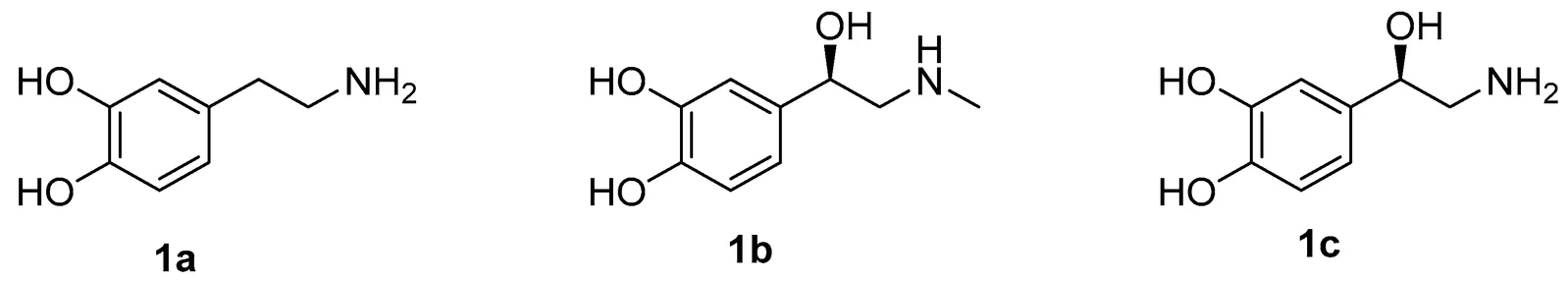
Chemical structures of dopamine (**1a**), epinephrine (**1b**), and norepinephrine (**1c**).

The electrochemical behavior of dopamine (**1a**, 50 μM), epinephrine (**1b**, 150 μM), and norepinephrine (**1c**, 150 μM) was investigated at GPH/SPE, TYR/GPH/SPE, and TYR/EHLNPs/GPH/SPE platforms by DPV in 0.1 M PBS (pH 7.0, containing 0.1 M KCl), recording the signal over a potential window from −0.1 V to 0.7 V. The chemical structures of the catecholamines investigated are reported in Figure 4. The concentrations of the catecholamines were selected based on their different enzymatic and electrochemical responses previously characterized at tyrosinase-based interfaces [10]. Since dopamine showed an approximately five-fold higher sensitivity and a greater apparent affinity toward tyrosinase than epinephrine and norepinephrine, a lower dopamine concentration (50 μM) was sufficient to obtain a well-defined and reproducible DPV response, whereas 150 μM was required for epinephrine and norepinephrine. These concentrations were therefore selected to provide clearly detectable reduction peaks for evaluating the cysteine-induced potential shifts, rather than for a direct comparison of current responses at equal analyte concentrations. The TYR/GPH/SPE and TYR/EHLNPs/GPH/SPE configurations were obtained by drop-casting of tyrosinase solution (0.5 mg/mL) onto bare GPH/SPE and EHLNPs/GPH/SPE surfaces, respectively, generating enzymatically modified interfaces.

**Figure 5:**
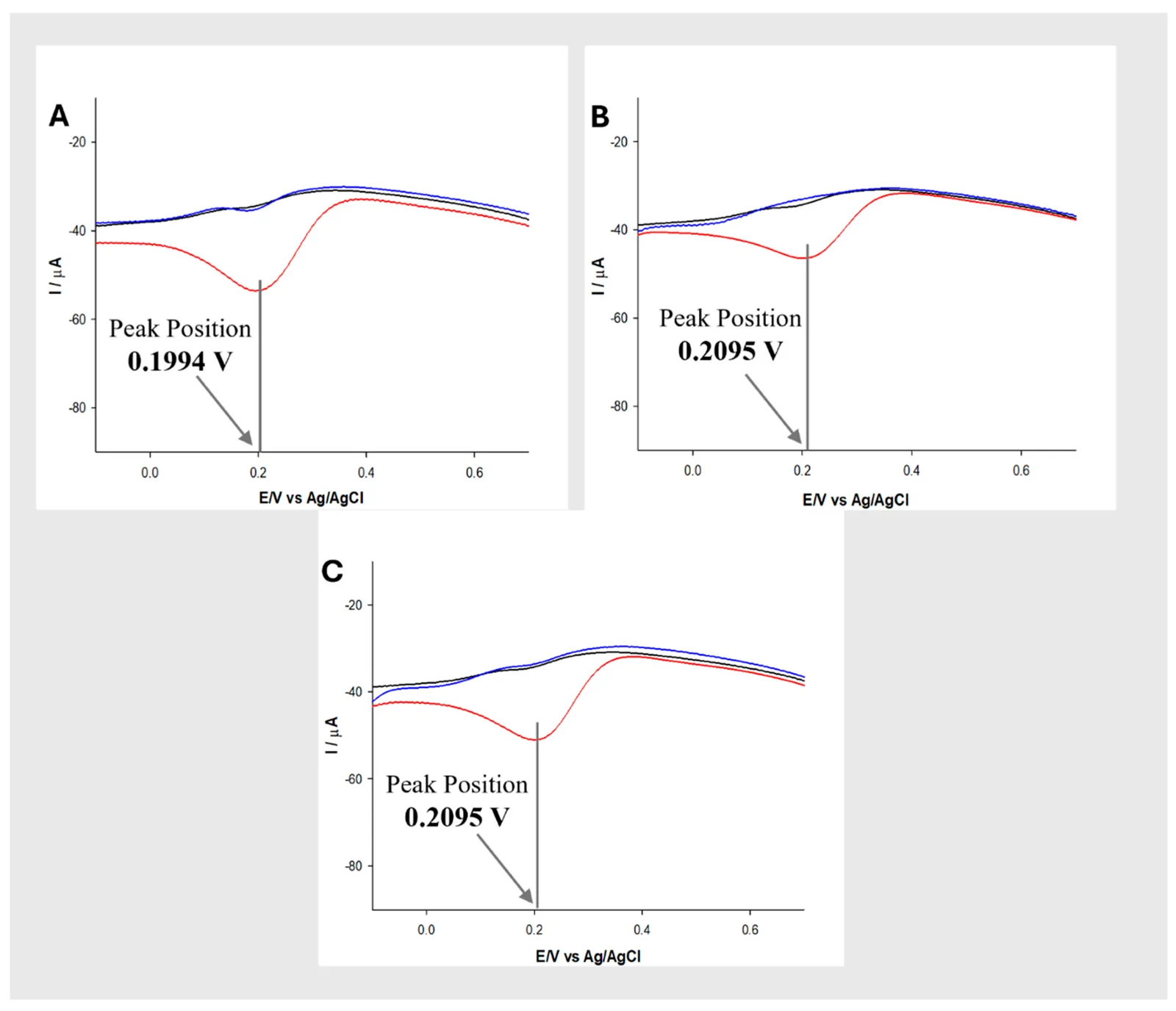
DPVs of (**A**) **1a** (50 μM), (**B**) **1b** (150 μM), and (**C**) **1c** (150 μM) recorded at GPH/SPE (black line), TYR/GPH/SPE (blue line), and TYR/EHLNPs/GPH/SPE (red line). Experimental conditions: 0.1 M PBS (pH 7.0, 0.1 M KCl); potential range −0.1 to 0.7 V.

As shown in Figure 5A–C, the GPH/SPE (black line) showed no significant electrochemical response. In a similar way, TYR/GPH/SPE (blue line) was characterized by weak and poorly defined electrochemical responses with no clear reduction peaks. The absence of detectable reduction signal suggests that tyrosinase was not efficiently immobilized on the electrode surface in the absence of EHLNPs. In contrast, TYR/EHLNPs/GPH/SPE (red line) exhibited well-defined reduction peaks for **1a**, **1b** and **1c** (Figure 5A–C), confirming the key role played by EHLNPs in promoting the immobilization and stabilization of tyrosinase at the electrode interface, enabling electron transfer and measurable electrochemical response [11]. The reduction peak potentials, defined as statistical means of six repeated measurements and associated standard deviation (SD), were 0.1994 ± 0.0004 V for **1a** (Figure 5A) and 0.2095 ± 0.0005 V for **1b** and **1c** (Figure 5B and Figure 5C, respectively). Thus, the three catecholamines showed very similar electrochemical responses, making their chemical discrimination challenging.

### 2.5. Electro-Biocatalytic Cysteine Reaction and Catecholamine Electrochemical Response

**Scheme 1:**
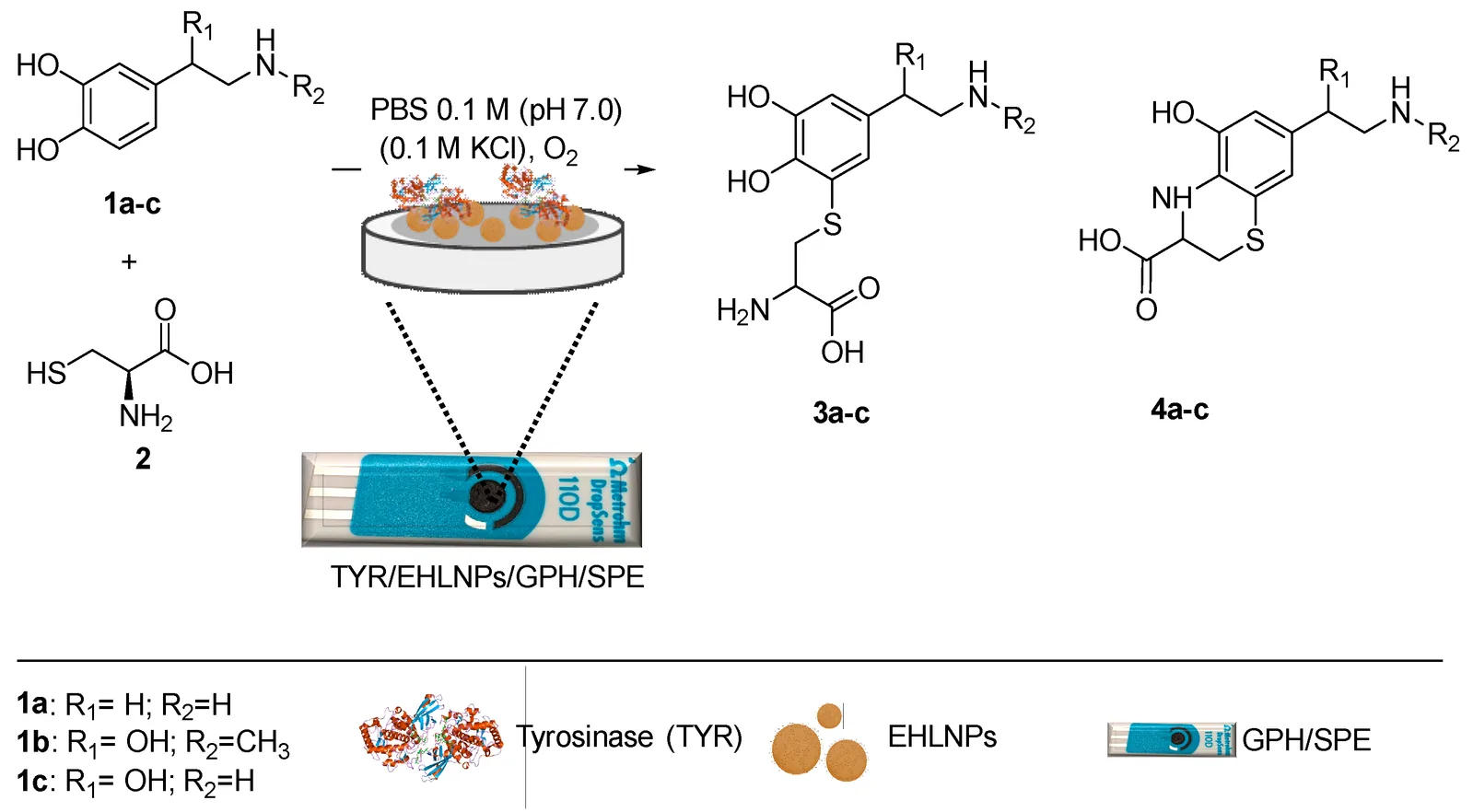
General behavior of the electro-biocatalytic oxidation of catecholamines **1a**–**1c** in the presence of cysteine **2** at the TYR/EHLNPs/GPH/SPE interface, leading to mono-cysteinyl **3a**–**3c** and **4a**–**4c**. Compounds **3c** and **4c** were identified in this study by HPLC-MS and HRMS analyses.

To overcome the similarity of the electrochemical responses of catecholamines, L-cysteine was used as nucleophile to synthesize bioconjugates by addition to quinone intermediates produced from immobilized tyrosinase. Scheme 1 represents some of the expected adducts based on previously reported studies [6,7,8,21,22,23,24].

**Figure 6:**
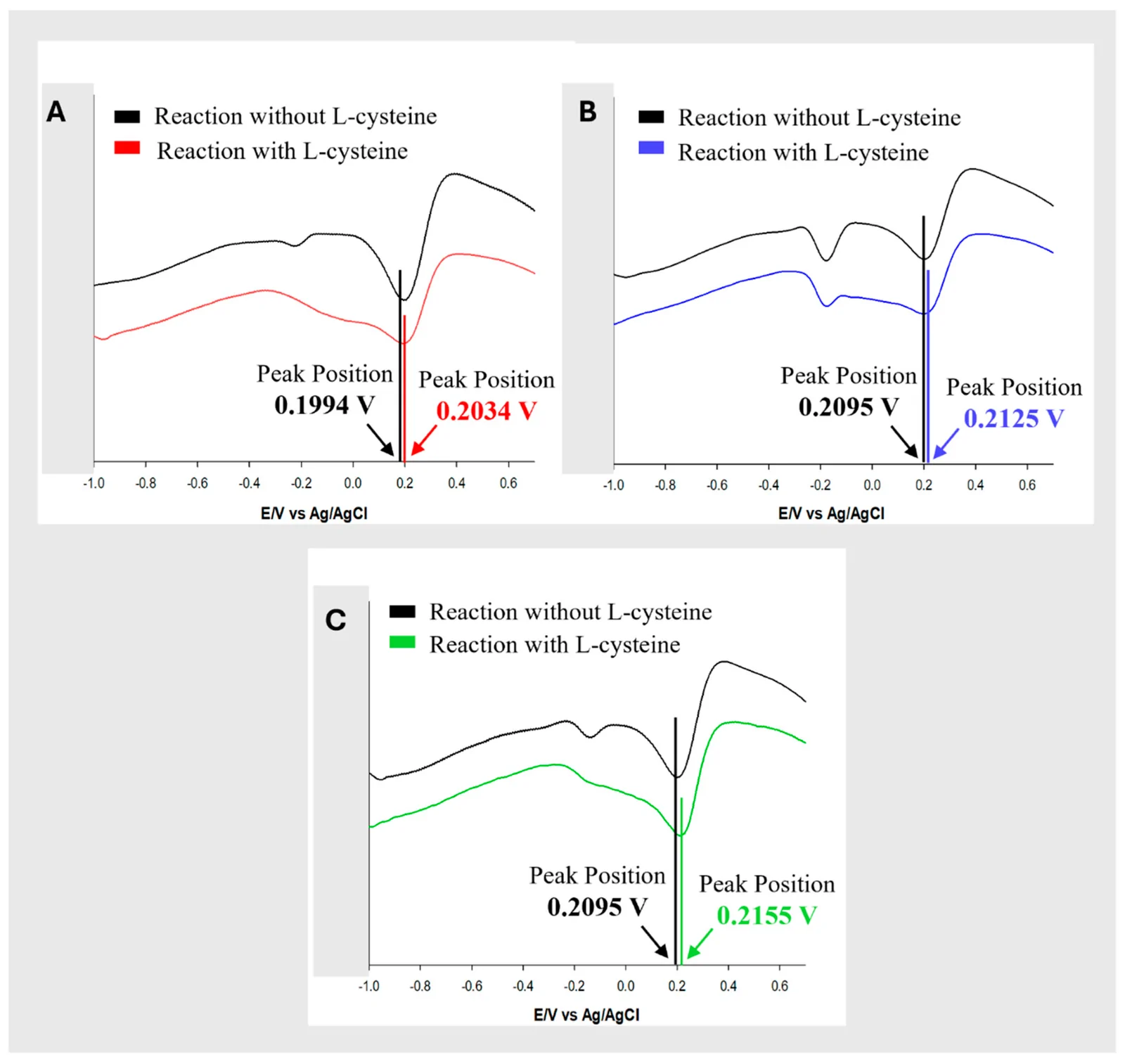
Panel (**A**): DPVs of **1a** (50 μM). Panel (**B**): DPVs of **1b** (150 μM). Panel (**C**): DPVs of **1c** (150 μM). Analyses were recorded at TYR/EHLNPs/GPH/SPE in the absence (black line) and presence of L-cysteine (red line for **1a**, blue line for **1b** and green line for **1c**). Experimental conditions: 0.1 M PBS (pH 7.0, 0.1 M KCl); potential range −0.1 to 0.7 V; L-cysteine in equimolar ratio (1:1) with catecholamine.

Electrochemical measurements were performed under the experimental conditions described above, in the presence of equimolar concentrations (1:1 ratio) of L-cysteine and selected catecholamine. Six measurements in replicate were repeated. Data is reported as a statistical means with associated SD. Figure 6 reports the comparison between DPV responses for each catecholamines in the presence and absence of cysteine. The reduction peak of **1a** shifted from 0.1994 ± 0.0004 V in the absence of L-cysteine to 0.2034 ± 0.0005 V in its presence (Figure 6A). A more pronounced shift was observed for **1b** (Figure 6B), where the reduction peaks potential moved from 0.2095 ± 0.0005 V to 0.2125 ± 0.0006 V. Similarly, **1c** (Figure 6C) exhibited an appreciable shift from 0.2095 ± 0.0004 V to 0.2155 ± 0.0005 V. These results demonstrated that L-cysteine was able to induce measurable changes in the reduction potentials of catecholamines.

To evaluate the possibility that the electrode may be modified after the reaction, it was recovered in the selected case of **1c**, and the reduction potential was measured again in the presence of norepinephrine and in the absence of L-cysteine under similar experimental conditions. The reduction potential was like the original value (0.2095 ± 0.0004 V) confirming that the electrode was not significantly modified.

**Figure 7:**
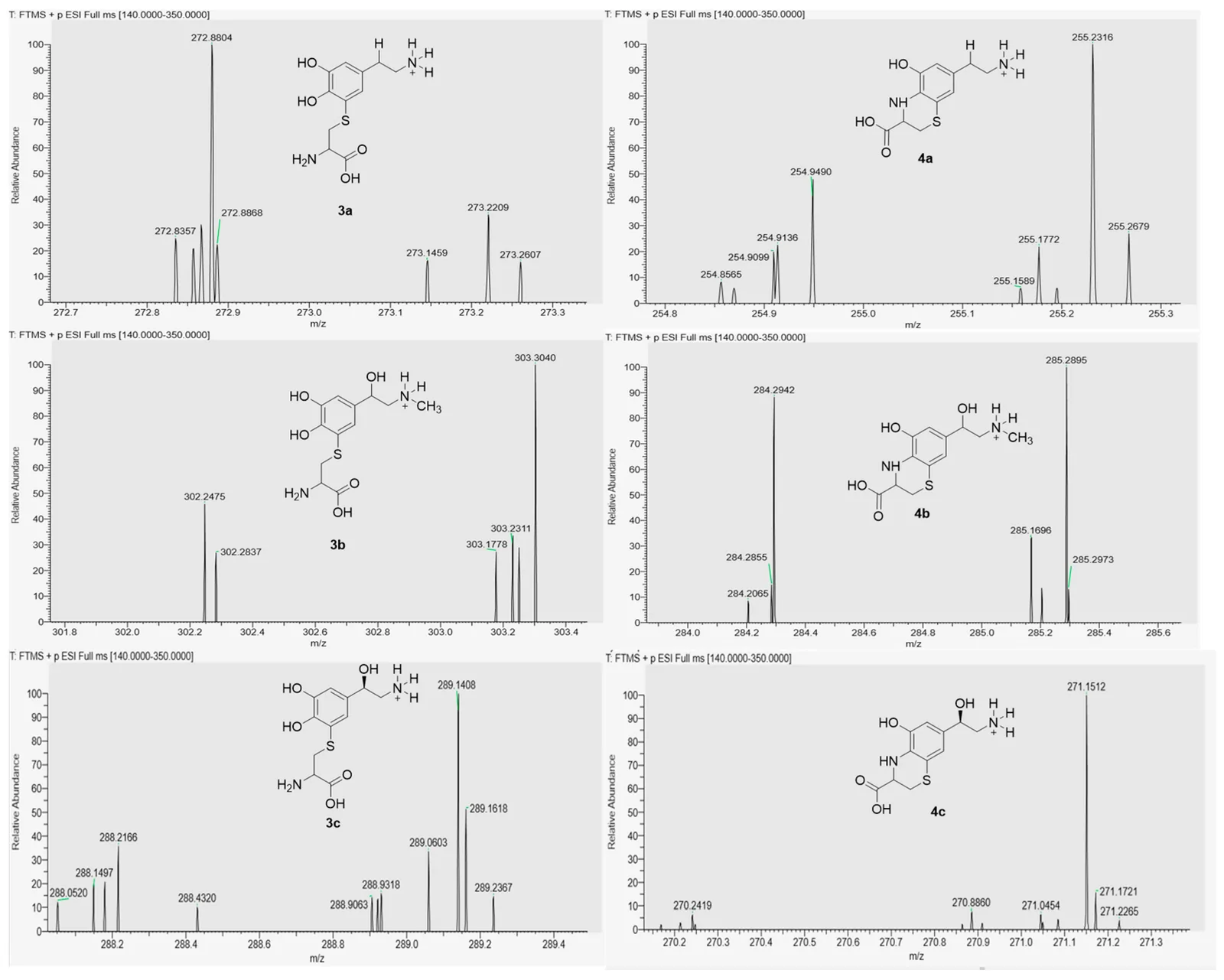
High-resolution mass spectrometry (HRMS) analysis of compounds **3a**–**4a, 3b**–**4b** and **3c**–**4c**.

High-performance liquid chromatography coupled with mass spectrometry (HPLC–MS) was employed to investigate the formation of cysteinyl adducts. The chromatographic profile of **1c** is reported in Figure S2. It showed a complex pattern, characterized by the presence of two major peaks localized at 12–13 min retention time. The peaks were assigned as the dihydrobenzothiazine monoadducts **3c** and **4c**, respectively (Figure S3). These compounds have been previously reported as well-established derivatives from the addition of L-cysteine to the quinone of norepinephrine [22]. Extensive mechanistic studies are described in the literature for quinone–thiol interaction [23,24]. In particular, **3c** showed the characteristic signal at *m*/*z* 144.93 consistent with a doubly charged ion [M + 2H] ^2+^ and correspondent to the expected molecular mass of 288 Da, while compound 4c displayed *m*/*z* 292.01, correspondent to the mono sodium coordination adduct [M + Na] ^+^ (molecular mass of 270 Da). The original spectra are in Figure S3. Compounds **3c** and **4c** have been previously detected under purely electrochemical conditions, where the oxidation of catecholamine was driven directly at the electrode surface [3,4]. The structure of **3c** and **4c** was further confirmed by high-resolution mass spectrometry (HRMS) analysis. As reported in Figure 7, the expected molecular mass of 288 Da and 270 Da were observed for **3c** and **4c**, respectively. HRMS analysis of similar adducts obtained from dopamine and epinephrine (compounds **3a**–**4a** and **3b**–**4b**) are reported in Figure 7.

## 3. Discussion

The reaction of dopamine (**1a**), epinephrine (**1b**), and norepinephrine (**1c**) with L-cysteine was realized by a novel electro-biocatalytic process occurring at the interface of functionalized TYR/EHLNPs/GPH/SPE thanks to the transformation of the substrates into distinct bioconjugates with cysteine as a nucleophile. This represents a proof-of-concept for the modulation of molecular structure and redox properties of similar compounds. The slight increase in electroactive area (A_E_), roughness factor (ρ), and heterogeneous electron transfer rate constant (k_0_) observed in the case of TYR/EHLNPs/GPH/SPE suggests that the NPs layer does not act as an insulating barrier. This behavior is consistent with the intrinsic aromatic nature of lignin nanoparticles, in which phenol subunits form ordered aggregates due to π–π interactions between HOMO and LUMO frontier molecular orbitals. This interaction facilitates electronic transfer processes [25]. Within this framework, EHLNPs also played the key role of supporting tyrosinase, enabling efficient immobilization and stabilization of tyrosinase and contemporary facilitation of the oxidation of the catecholamines. In the absence of this organized interface, the system remained electrochemically silent. Notably, while catecholamines exhibited very similar electrochemical responses in the absence of L-cysteine, the introduction of this nucleophile led to an appreciable shift in the reduction peak potential. This behavior was a consequence of the formation of catecholamine–cysteine adducts with different redox properties through quinone–thiol coupling reactions occurring at the bio-electrochemical interface. Even if the difference in the observed reduction potential is not very high, it is statistically significant and, in principle, it is a proof-of-concept of the reliability of this combined approach to identify different catecholamines. HPLC–MS and HRMS analyses confirmed the formation of representative monoadducts in the case of catecholamines, supporting the interpretation of the reaction pathway. The oxidation of catecholamine and nucleophile addition afforded an overall controlled reactivity, preventing the possible electrode fouling which is typically observed under direct electrochemical conditions working in the absence of the enzymatic control [26]. In addition, since the primary oxidation step is enzymatically driven, the electrochemical process works at significantly lower applied potentials compared to direct electrochemical oxidation, enhancing the sustainability of the process [27]. This feature minimizes undesired side reactions and enhances both selectivity and operational stability under mild conditions. On the other hand, the observed low difference in DPV values limit practical applications in the differentiation of catecholamines and require further optimization studies. Overall, the present study represents proof-of-concept about the use of reaction-driven differentiation of similar catecholamines [28].

## 4. Materials and Methods

### 4.1. Materials

Tyrosinase (EC 1.14.18.1, 8503 U mg^−1^, derived from mushroom, TYR), dopamine hydrochloride, epinephrine, norepinephrine, and L-cysteine were obtained from Sigma-Aldrich (St. Louis, MO, USA). Enzymatic hydrolysis lignin (EHL) was obtained from Chemical Point. The phosphate-buffered solution (PBS) was prepared using Na_2_HPO_4_ and NaH_2_PO_4_ salts, while pH adjustments were carried out by addition of NaOH or HCl as required. Screen-printed electrodes (SPEs), including carbon (SPCE), gold (Au-SPE), multi-walled carbon nanotubes (MWCNTs/SPE), and graphene-based electrodes (GPH/SPE), used for electrode selection and platform fabrication, were supplied by Dropsens (Metrohm Dropsens, Asturias, Spain).

### 4.2. Preparation of EHLNPs

Hybrid melanin lignin nanoparticles were prepared from enzymatic hydrolysis lignin (EHL) by nanoprecipitation. Briefly, EHL (170 mg) was dissolved in the ternary mixture (primary solvent) water (22% *v*/*v*, 0.54 mL), ethanol (39% *v*/*v*, 0.98 mL) and THF (39% *v*/*v*, 0.98 mL) was added to deionized water (12.5 mL) as antisolvent under gentle mechanical stirring to yield EHLNPs isolated by centrifugation and freeze dry.

### 4.3. Dynamic Light Scattering (DLS) and ζ-Potential Measurements

The dynamic light scattering (DLS) and electrophoretic light scattering (ELS) measurements were performed using a Zetasizer Advance Series Pro (Malvern Panalytical, Malvern, Worcestershire, UK) equipped with a 633 nm red laser operating at a fixed backscattering angle of 173°. Nanoparticle suspensions were prepared in deionized water at a concentration of 1 mg/mL and sonicated prior to analysis. The hydrodynamic diameter (Z-average) and polydispersity index (PDI) were derived from cumulant analysis, while the ζ-potential was calculated from electrophoretic mobility using the Smoluchowski approximation.

### 4.4. Field-Emission Scanning Electron Microscopy (FE-SEM)

FE-SEM was performed by FESEM ZEISS GeminiSEM500 (Carl Zeiss Microscopy GmbH, Jena, Germany) at 5 kV. The sample (20 μL in deionized water) was dropped on specimen stubs, dried air, and coated with gold by sputtering with an AGAR (Auto Sputter Coater). Before the measurement, the sample received a deposition of chromium thin film (5 nm) by sputter coating using a QUORUM Q 150T ES plus coater (Laughton, East Sussex, UK).

### 4.5. ^31^P NMR

The quantitative analysis of hydroxyl groups of EHL was conducted by ^31^P NMR analysis. Briefly, EHL (30 mg) were treated with 2-chloro-4,4,5,5-tetramethyl-1,3,2-dioxaphospholane (0.9 mmol) in the presence of N-hydroxy-5-norbornene-2,3-dicarboxylic acid mine (0.010 mmol) as an internal standard and chromium (III)acetylacetonate as relaxation agent. ^31^P NMR spectra were recorded on a Bruker (400 MHz).

### 4.6. Electrochemical Measurements and Platform Fabrication

Electrochemical measurements were carried out in a thermostatically controlled 10 mL glass cell equipped with a conventional three-electrode system (model 6.1415.150, Metrohm, Herisau, Switzerland). Screen-printed electrodes (SPEs) were used as working electrodes, an external Ag/AgCl/KClsat electrode (198 mV vs. NHE; cat. 6.0726.100, Metrohm, Herisau, Switzerland) as the reference electrode, and a glassy carbon rod (cat. 6.1248.040, Metrohm, Herisau, Switzerland) as the counter electrode. All measurements were performed using an Autolab potentiostat/galvanostat (Eco Chemie, Utrecht, The Netherlands). For the preliminary electrochemical characterization, different screen-printed electrodes (SPCE, Au-SPE, MWCNTs/SPE, and GPH/SPE) were modified with EHLNPs by drop-casting 6 μL of a 1 mg/mL aqueous suspension onto the electrode surface, followed by drying at room temperature (1 h). The resulting EHLNPs-modified electrodes were used to evaluate the electrochemical performance of the different platforms and to determine the corresponding electrochemical parameters by cyclic voltammetry (CV) in a 2.5 mM [Fe(CN)_6_]^3−^/^4−^ solution containing 0.1 M KCl. For the selective detection of catecholamines, the GPH/SPE, identified as the optimal platform, was further modified with tyrosinase by drop-casting 6 μL of a 0.5 mg/mL solution (in 0.1 M PBS, pH 7.0, containing 0.1 M KCl), followed by drying at room temperature (1 h), obtaining the TYR/EHLNPs/GPH/SPE configuration. Electrochemical measurements were carried out in 0.1 M PBS (pH 7.0, containing 0.1 M KCl) by differential pulse voltammetry (DPV) in the presence of dopamine (50 μM), epinephrine (150 μM), and norepinephrine (150 μM). In experiments performed in the presence of L-cysteine, a 1:1 molar ratio between L-cysteine and each catecholamine was used. All measurements were performed in six replications. Limit of detection and limit of quantification are reported as a selected case for dopamine in Table S2. Note that TYR/EHLNPS/GPH/SPE prepared in different batches worked in a similar way.

### 4.7. Liquid Chromatography–Mass Spectrometry (LC–MS) and High-Resolution Mass Spectrometry (HRMS)

The HPLC-MS analysis was performed using an Ultra high-performance liquid chromatography system Vanquish coupled with MS, Thermo Scientific (Waltham, MA, USA) equipped with Avantor ACE^®^ reverse-phase C18 analytical column (RP18, ODS, Octadecyl, 5 µm, 100 Å, 4.6 × 250 mm). The analysis was performed using Acetonitrile (MeCN) (A) and water (0.1% *v*/*v* TFA) (B) as the mobile phase at a flow rate of 0.8 mL min^−1^. The gradient used was: 10% MeCN for 10 min, a linear gradient to 70% MeCN over 10 min, 100% MeCN for 5 min, followed by 10% MeCN for 10 s. The column temperature was maintained at 25 °C and the total analysis time was 30 min to ensure complete elution and column re-equilibration. Detection was measured at 254 nm using a UV/Vis detector.

High-resolution mass spectrometry (HRMS) analyses were performed using a Vanquish UHPLC system (Germering, Germany) coupled to an Exploris 120 Orbitrap mass spectrometer (Bremen, Germany). Chromatographic separation was achieved on a Hypersil GOLD column (2.1 × 100 mm, 1.9 μm particle size). The mobile phase consisted of (A) water containing 0.1% trifluoroacetic acid (TFA) and (B) acetonitrile. The flow rate was set at 0.5 mL min^−1^, and the column temperature was maintained at 25 °C. A gradient elution program was applied as follows: 2% B at 0 min, held for 4 min; increased to 45% B at 14 min; ramped to 95% B at 18 min and held for 3 min; subsequently returned to 2% B at 21 min and equilibrated for 4 min. The total running time was 25 min. The mass spectrometer was operated in full scan mode with a resolving power of 120 K at *m*/*z* 200 and a mass accuracy within 5 ppm. Data were acquired in positive ion mode over a scan range of *m*/*z* 140–350. The ion source was a heated electrospray ionization (H-ESI) source operated with the following parameters: spray voltage 3500 V, sheath gas 35 (Arb), auxiliary gas 10 (Arb), sweep gas 1 (Arb), ion transfer tube temperature 320 °C, vaporizer temperature 280 °C, and RF lens set to 70%.

## Figures and Tables

**Table 1:** Electrochemical parameters of bare and EHLNPs-modified SPEs: peak-to-peak separation (ΔE) ^[a]^, anodic peak current (I_pa_) ^[b]^, and cathodic peak current (I_pc_) ^[c]^.

Entry	Platform	ΔE ^[a]^ (mV)	I_pa_ ^[b]^ (μA)	I_pc_ ^[c]^ (μA)
1	SPCE	210	+50	−45
2	MWCNTs/SPE	127	+63	−62
3	Au-SPE	91	+75	−72
4	GPH/SPE	98	+63	−61
5	EHLNPs/SPCE	392	+28	−22
6	EHLNPs/MWCNTs/SPE	196	+41	−39
7	EHLNPs/Au-SPE	148	+49	−45
8	EHLNPs/GPH/SPE	91	+69	−68

^[a]^ ΔE (peak-to-peak separation). ^[b]^ I_pa_ (anodic peak current). ^[c]^ I_pc_ (cathodic peak current).

**Table 2:** Electrochemical parameters of GPH/SPE and EHLNPs/GPH/SPE platforms: A_E_ ^[a]^, ρ ^[b]^, and k0 ^[c]^. Experimental conditions: 2.5 mM [Fe(CN)_6_]^3−^/^4−^ in 0.1 M KCl solution.

Entry	Platform	A_E_ ^[a]^ (cm^2^)	ρ ^[b]^	k_0_ ^[c]^ 10^−3^ (cm s^−1^)
1	GPH/SPE	0.19 ± 0.002	1.74	2.32 ± 0.4
2	EHLNPs/GPH/SPE	0.22 ± 0.003	2.04	2.51 ± 0.5

^[a]^ A_E_ (electroactive area). ^[b]^ ρ (roughness factor). ^[c]^ k0 (heterogeneous electron transfer rate constant).

## Data Availability

The original contributions presented in the study are included in the article. Further inquiries can be directed at the corresponding author.

## Supplementary Materials

The following supporting information can be downloaded at: https://www.mdpi.com/article/10.3390/molecules31162839/s1, Table S1: Functional group distribution by quantitative ^31^P NMR analyses of EHL; Figure S1: Dynamic light scattering (DLS) of EHLNPs; Figure S2: Representative HPLC–UV chromatogram (λ = 254 nm) of the ethyl acetate extract obtained after the electrochemical treatment of norepinephrine in the presence of cysteine; Figure S3: Mass spectrometric analysis of the main products detected in the reaction between norepinephrine and cysteine; Table S2: Linear Range, LOD, LOQ and R^2^ of dopamine with TYR/EHLNPS/GPH/SPE.

## Abbreviations

The following abbreviations are used in this manuscript:

A_E_	Electroactive area
Au-SPE	Gold screen-printed electrode
CV	Cyclic voltammetry
DLS	Dynamic light scattering
DPV	Differential pulse voltammetry
EHL	Enzymatic hydrolytic lignin
EHLNPs	Enzymatic hydrolytic lignin nanoparticles
FE-SEM	Field-emission scanning electron microscopy
GPH	Graphene
H-ESI	Heated electrospray ionization
HOMO	Highest occupied molecular orbital
HPLC-MS	High-performance liquid chromatography–mass spectrometry
HRMS	High-resolution mass spectrometry
I_pa_	Anodic peak current
I_pc_	Cathodic peak current
K_0_	Heterogeneous electron transfer rate constant
LC-MS	Liquid chromatography–mass spectrometry
LNPs	Lignin nanoparticles
LUMO	Lowest unoccupied molecular orbital
MWCNTs	Multi-walled carbon nanotubes
NMR	Nuclear magnetic resonance
PBS	Phosphate-buffered solution
PDI	Polydispersity index
SD	Standard deviation
SPCE	Screen-printed carbon electrode
SPE	Screen-printed electrode
TFA	Trifluoroacetic acid
THF	Tetrahydrofuran
TYR	Tyrosinase
UHPLC	Ultra-high-performance liquid chromatography
UV-Vis	Ultraviolet–visible
ΔE	Peak-to-peak separation
Ρ	Roughness factor
