# Supplementary material for: Electro-Biocatalytic Reactivity of Catecholamine at a Lignin Nanoparticle–Tyrosinase Interface
Source: Molecules. 2026 Aug 14;31(16):2839. doi: 10.3390/molecules31162839 (PMC13515523; doi:10.3390/molecules31162839)
Supplement: Supplementary file 1 [file molecules-31-02839-s001.zip › molecules-4458163-supplementary.pdf]

Quantitative ^{31}P NMR analysis of EHL

Table S1. Functional group distribution by quantitative ^{31}P NMR analyses of EHL.

**Lignin sample**	**EHL**
**Lignin Hydroxyl Group[a]**	**Abundance (mmol/g)[a]**
Aliphatic OH	1.639
C_5 substituted/condensed OH	1.466
guaiacyl OH	1.615
p-hydroxyphenyl OH	1.108
carboxylic acid OH	0.966

The ^{31}P NMR have been performed in pyridine for 2 hours at room temperature.

Dynamic Light Scattering (DLS) and ζ-Potential Measurements

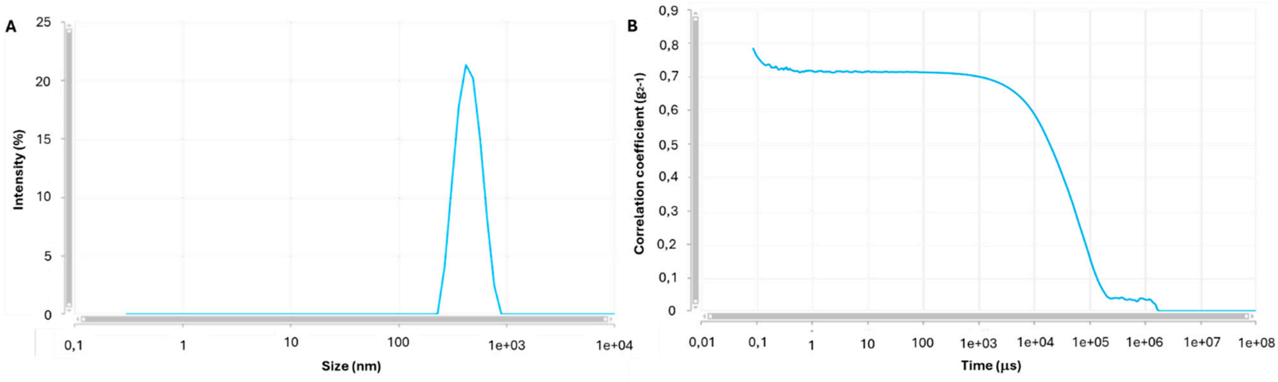

Figure S1. Dynamic light scattering (DLS) of EHLNPs. (A) Intensity-weighted hydrodynamic size distribution of EHLNPs. (B) Corresponding intensity autocorrelation function used for size determination.

Liquid Chromatography–Mass Spectrometry (LC–MS)

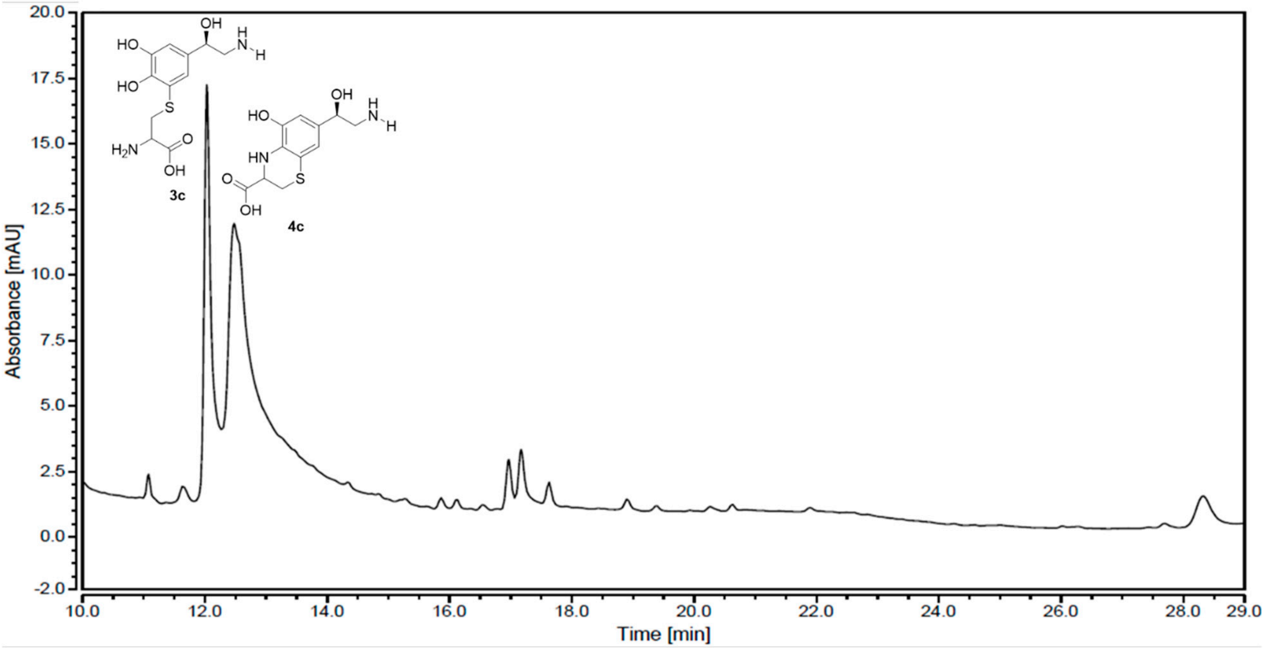

Figure S2. Representative HPLC–UV chromatogram (λ = 254 nm) of the ethyl acetate extract obtained after the electrochemical treatment of norepinephrine in the presence of cysteine. The chromatogram is shown as a magnified view

of the 10–20 min retention time window to highlight the region containing the main reaction products. Two major peaks at t_n = 12.03 min and 12.48 min were assigned to dihydrobenzothiazine monoadducts 3c and 4c, respectively.

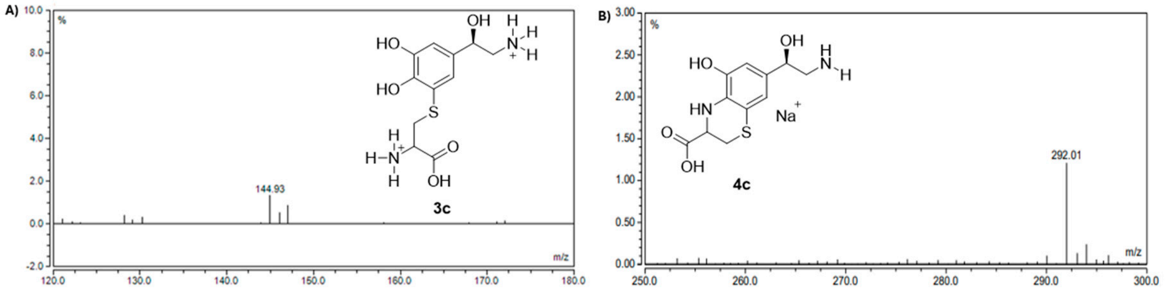

Figure S3. Mass spectrometric analysis of the main products detected in the reaction between norepinephrine and cysteine. Panel A shows the mass spectrum associated with compound 3c, displaying a characteristic signal at m/z 144.93, consistent with a doubly charged ion ($[M + 2H]^{2+}$) corresponding to an expected molecular mass of 288 Da. Panel B shows the mass spectrum of compound 4c, with a dominant signal at m/z 292.01, attributed to the sodium adduct $[M + Na]^+$ of a species with expected molecular mass of 270 Da. The formation of sodium adducts is commonly observed in positive electrospray ionization (ESI) conditions, resulting in a characteristic +23 Da shift relative to the neutral molecule.

Limit of detection (LOD), limit of quantification (LOQ) and equation of the calibration curve

The calibration curve was obtained by amperometric measurements using the Tyr/EHLNPs/GPH/SPE platform under optimal experimental conditions. Briefly, successive aliquots of dopamine were added to the electrochemical cell, and the corresponding steady-state current variations (ΔI) were measured after each addition. The calibration plot was constructed by plotting the current variation (ΔI, µA) as a function of dopamine concentration (from 8 to 52 µM), yielding the linear regression equation $y = 0.0335x + 0.0340$ (R^2 = 0.9958), where y is the current variation (ΔI, µA) and x is the dopamine concentration (µM). The limit of detection (LOD) and the limit of quantification (LOQ) were calculated according to LOD = $3\sigma/m$ and LOQ = $10\sigma/m$, where m is the slope of the calibration curve and σ is the standard deviation of the linear regression. LOD and LOQ are reported in Table S2.

Table S2. Linear Range, LOD, LOQ and R^2 of dopamine with TYR/EHLNPS/GPH/SPE.

Platform	Linear Range (µM)	LOD (µM)	LOQ (µM)	R^2
TYR/EHLNPS/GPH/SPE	8-27	1.6	5.6	0.9960
